# Neuroprotective effects of quercetin on hippocampal CA1 neurons following middle cerebral artery ischemia‒reperfusion in male rats: a behavioral, biochemical, and histological study

**DOI:** 10.1186/s12883-024-04017-z

**Published:** 2025-01-06

**Authors:** Mehran Mahyar, Erfan Ghadirzadeh, Pedram Nezhadnaderi, Zahrasadat Moayedi, Parniyan Maboud, Arvin Ebrahimi, Ali Siahposht-Khachaki, Narges Karimi

**Affiliations:** 1 Department of Neurology, Mazandaran University of Medical Sciences, Sari, Iran; 2 Gastrointestitional Cancer Research Center, Non-Communicable Disease Institute, Mazandaran University of Medical Sciences, Sari, Iran; 3 Student Research Committee, Shiraz University of Medical Sciences, Shiraz, Iran; 4 Department of Internal Medicine, School of Medicine, Shiraz University of Medical Sciences, Shiraz, Iran; 5 Department of Pharmacology Sciences, School of Pharmacy, Mazandaran University of Medical Sciences, Ramsar, Iran; 6 Immunogenetics Research Center, Department of Physiology, Mazandaran University of Medical Sciences, Sari, Iran; 7 Department of Neurology, School of Medicine, Immunogenetic Research Center, Mazandaran University of Medical Sciences, Sari, Iran

**Keywords:** Quercetin, Cerebral ischemia, Neuroprotection, Focal cerebral ischemia, Middle cerebral artery occlusion

## Abstract

**Introduction:**

Cerebral ischemic strokes cause brain damage, primarily through inflammatory factors. One of the regions most affected by middle cerebral artery occlusion (MCAO) is the hippocampus, specifically the CA1 area, which is highly susceptible to ischemia. Previous studies have demonstrated the anti-inflammatory properties of quercetin. Therefore, this study aimed to investigate the neuroprotective effects of quercetin on hippocampal CA1 neurons following MCAO.

**Materials and methods:**

Fifty-six male Albino Wistar rats were divided into seven groups (intact, sham, stroke, vehicle, and three quercetin-treated groups receiving 5, 10, and 20 mg/kg, respectively), each containing 8 rats. Various assessments, including brain water content, the rotarod test, the Bederson neurological score, the Morris water maze (MWM) test, the shuttle box test, histopathological evaluations, and measurements of interleukin-10 (IL-10) and interleukin-1β (IL-1β) levels, were conducted across the groups.

**Results:**

Compared with control rats, 5 and 10 mg/kg quercetin-treated rats presented significant improvements in brain water content, neurological function, and motor function and improved performance in the MWM and shuttle box tests. Histopathological analyses revealed better preservation of CA1 neurons in these groups. Additionally, IL-10 levels significantly increased, whereas IL-1β levels significantly decreased. However, the group receiving 20 mg/kg quercetin showed no statistically significant changes in the parameters assessed (*P* > 0.05).

**Conclusion:**

Quercetin may help prevent or ameliorate brain injuries caused by acute stroke, suggesting its neuroprotective effects. The reduction in IL-1β and increase in IL-10 may play key roles in quercetin’s protective mechanism.

## Background

Stroke, defined as sudden neurological damage caused by impaired blood perfusion to the brain, is one of the leading causes of death and disability worldwide [1]. Among the various forms of stroke, ischemic stroke, caused by an obstruction in a blood vessel supplying the brain, is the most prevalent [2]. The middle cerebral artery (MCA) is one of the most commonly affected vessels in ischemic stroke, leading to significant neurological deficits [3]. The hippocampus, particularly the CA1 region, is highly vulnerable to ischemic injury because of its high metabolic demand and sensitivity to oxygen and glucose deprivation [4]. This vulnerability can result in severe cognitive and behavioral impairments, making it a critical region for studying the effects of ischemic stroke [5]. Thus, understanding the mechanisms that protect or damage the CA1 region during ischemic events is crucial for developing effective neuroprotective strategies.

Ischemia‒reperfusion injury occurs when the blood supply returns to the tissue after a period of ischemia or a lack of oxygen [6]. Paradoxically, the restoration of circulation exacerbates cellular injury and inflammation, contributing to neuronal death and functional deficits [6, 7]. To mitigate these effects, neuroprotective agents such as mexiletine have been extensively studied [8]. Quercetin (QC), a naturally occurring flavonoid found in many fruits and vegetables, is a promising agent [9].

QC is well known for its potent antioxidant, anti-inflammatory, and antiapoptotic properties. Recent studies have demonstrated its potential in reducing oxidative stress and neuronal damage in various models of neurodegeneration and global cerebral ischemia‒reperfusion injury [10]. However, its effects on regional brain ischemia‒reperfusion injury, particularly in hippocampal CA1 neurons following MCA occlusion (MCAO), remain underexplored.

Thus, this study aimed to investigate the neuroprotective effects of QC on hippocampal CA1 neurons in a rat model of MCAO by examining behavioral outcomes, biochemical markers of inflammation (interleukins), and histological changes.

## Materials and methods

### Animals

This experimental study was performed on 56 male Albino Wistar rats (250–300 g, and 8–10 weeks old). The animals were housed with free access to food and water, maintained at a temperature of 22–25 °C, and subjected to a 12:12-hour light‒dark cycle. This study was approved by the Research Ethics Committee of Laboratory Animals, Mazandaran University of Medical Sciences (Ethics Approval Code: IR.MAZUMS.AEC.1401.046). All procedures performed in this study were in accordance with the ARRIVE guidelines and the ethical standards of the Institutional Research Ethics Committee of Mazandaran University of Medical Sciences and in accordance with the Guidance on the Operation of the Animals (Scientific Procedures) Act 1986 and associated guidelines.

### Experimental groups

The rats were randomly divided into the following seven experimental groups (8 rats in each group):

1. Intact group: No interventions.
2. Sham group: Underwent surgery without MCAO induction.
3. Stroke group: MCAO was induced after anesthesia.
4. Solvent group: MCAO + 0.1% DMSO.
5. Low-dose group: MCAO + 5 mg/kg QC.
6. Medium-dose group: MCAO + 10 mg/kg QC.
7. High-dose group: MCAO + 20 mg/kg QC.

All the injections (QC, DMSO, Ketamine, Xylazine, and Medetomidine) were administered intraperitoneally (IP) (except Midazolam which was administered subcutaneously) 30 min after stroke induction and were repeated every 24 h for up to three days (four total doses in the following hours after MCAO induction: 0.5, 24, 48, and 72).

### Inclusion criteria

- Successful performance in pretest exams confirming neurological deficits consistent with MCAO.
- Confirmation of ischemic brain lesion using triphenyl tetrazolium chloride (TTC) staining.

### Exclusion criteria

- Animals not passing pretest exams.
- Failure to exhibit clear neurological deficits post-surgery (indicating unsuccessful MCAO induction).
- Absence of TTC-confirmed brain lesions.
- Severe post-operative complications (e.g., infection or hemorrhage) resulting in animal welfare concerns and bias in study results.
- Mortality occurring before the endpoint of the experiment.

### MCAO Model

The intraluminal filament technique was used to induce MCAO [11, 12]. The rats were anesthetized with ketamine (50 mg/kg) and xylazine (10 mg/kg), along with midazolam (2 mg/kg subcutaneously) and medetomidine (0.1 mg/kg) for additional analgesia. To induce ischemia, a 2 cm incision was made in the neck to expose the common carotid artery (CCA), and the occipital and superior thyroid arteries were cauterized. The external carotid artery (ECA) was permanently sutured, while the CCA and internal carotid artery (ICA) were temporarily clamped. A small incision was made in the ECA to insert a nylon 3/0 monofilament. The nylon 3/0 monofilament was advanced 18–20 mm into the ICA to occlude the MCA and induce ischemia. After one hour, the filament and CCA clamp were withdrawn to restore blood flow. The incision was closed, and the animals were allowed to recover from anesthesia.

### Neurological assessment

The neurological outcomes were assessed with the Bederson rating system at 24 and 48 h post-MCAO [13]. This system is a standardized method for evaluating the severity of neurological deficits following MCAO in animal models. The Bederson scale ranges from 0 to 4, with higher scores indicating more severe neurological impairments. The scoring criteria are as follows:

- *Score 0*: No neurological deficit.
- *Score 1*: Flexion of the contralateral forelimb when the animal is suspended by the tail.
- *Score 2*: Decreased resistance to lateral push (mild circling behavior).
- *Score 3*: Severe circling behavior due to loss of postural control.
- *Score 4*: Loss of spontaneous motor activity or inability to walk.

### Motor function

A rotarod device (6700 MT model, Borjsanat Company, IR) was used to assess motor coordination and balance in rats 24 and 48 h after MCAO induction. The device features a rotating rod with adjustable speeds from 0 to 40 rpm. Initially, the rats were familiarized with the device by placing it on a rotating rod at 10 rpm with an acceleration of 7 rpm^2^. Following this, a balance test was conducted 30 min after the training session to assess their ability to remain on the rod. Only animals that demonstrated the ability to remain on the rod for at least 100s by the final day of training were included in the study. One hour post QC administration, the rats were placed on a rotarod, and the duration with which they maintained balance and resisted movement was recorded. The maximum time allowed for each animal in this test was 300s [14].

### Morris water maze (MWM)

The MWM [15, 16] is a cylindrical metal tank (140 cm diameter, 55 cm height) filled with 20 ± 1 °C water to a depth of 25 cm that is employed to assess spatial learning and memory. A hidden platform (11 cm diameter) was placed 1 cm below the water surface in one quarter. The rats’ movements were recorded by a camera to measure the following variables via EthoVision XT 10.1 software (Noldus Information Technology, Netherlands): swimming speed and the percentage of time spent in the target quarter. Twenty-four hours before training, the rats were placed in the tank without the platform for 1 min. The training was conducted over 2 days, with 4 trials each day. The platform remained in the center of one quarter. The rats were released from one of four starting points (north, south, east, or west) facing the tank wall, with the starting points randomized. Each trial ended when the rat found the platform or after 60s. The rats rested for 50 s between trials (20 s on the platform, 30 s off). The rats that failed to find the platform within 60 s were guided to it. After the trials, the rats were dried and returned to their cages. Twenty-four hours after training, the platform was removed, and the rats were released from a starting point. Behavior was recorded for 60s to measure the percentage of time spent in the target quadrant. Fifteen minutes after the probe test, the visible platform test was conducted. The platform, covered with aluminum foil, was placed in the center of a different quarter. This test assesses the potential effects of drug intervention on visual‒motor and motivational abilities [17].

### Shuttle box device

The shuttle box is a two-compartment plexiglass box designed for passive avoidance learning (PAL) studies. Each compartment measured 20 × 40 × 20 cm. One compartment is brightly lit by a 100-watt lamp positioned 40 cm above, while the other is dark. A guillotine door (5.7 × 5.7 cm) separates the two compartments. The floors of both compartments consisted of 2 mm stainless steel rods spaced 1 cm apart. The floor of the dark compartment is connected to an electrical circuit, enabling the delivery of a controlled electric shock to the animal. To acclimate the animal to the shuttle box, the animal was placed in the bright compartment. After 10 s, the guillotine door was lifted. As soon as the animal’s rear legs entered the dark compartment, the door was closed. After 30s in the dark compartment, the animal was returned to its cage. This process was repeated 30 min later, and the time the animal stayed in the bright room before entering the dark room (step-through latency, STL) was recorded. Animals with an STL longer than 60s were excluded because of low motivation for the dark room. Recordings were quantified via EthoVision XT 10.1 software (Noldus Information Technology, Netherlands).

Thirty minutes after the second acclimation, the animal was placed in the bright room for 10s, after which the guillotine door was opened. Upon entering the dark compartment, the door was closed, and an electric shock (50 Hz, 1 amp, 1.5 s duration) was delivered through the floor. After 20s, the animal was returned to its cage. After 2 min, the animal was reintroduced to the bright room. If the animal avoided the dark room for 120s, it was considered to be a successfully learned PAL. If the animal re-entered the dark room, it was shocked again, and the number of training sessions required for a successful PAL was recorded.

Twenty-four hours after training, the animal was placed in the bright compartment for 10s, after which the guillotine door was opened. The time taken for the animal to enter the dark compartment (STL after training) was recorded. The animal’s behavior was monitored for 300s, and the STL, total time spent in the dark compartment, and number of entries into the dark compartment were recorded. In this study, the STL after training and the total time spent in the dark compartment were the primary measures used for analysis. These metrics assess the effectiveness of the learning and retention process, indicating an animal’s ability to remember and avoid the compartment associated with an unpleasant stimulus (electric shock) [18].

### Collection of Cerebrospinal Fluid (CSF) samples from Cisterna magna

First, the animals were anesthetized with ketamine (50 mg/kg) and xylazine (10 mg/kg), their heads were fixed in a stereotaxic frame, and their neck hair was shaved. With the animal’s head bent at 45 degrees, a polyethylene tube and Hamilton syringe were used to extract 200 µl of CSF from the Cisterna magna, which is located between the posterior bone and the cervical atlas [19]. The CSF fluid was carefully collected to avoid blood contamination, transferred to an Eppendorf tube, and immediately fixed in liquid nitrogen.

### Measuring interleukins in CSF

In this study, interleukin 10 (IL-10) and IL-1β concentrations in CSF were evaluated via enzyme‒linked immunosorbent assay (ELISA) of the collected samples via rat IL-10 and IL-1β ELISA kits (MBS2702038 and MBS2707969) from MyBiosource, Inc. (San Diego, CA, USA) according to the manufacturer’s instructions.

### Histological assessment

For histopathological evaluation, brain samples from each group were obtained under euthanasia induced by sodium thiopental (60 mg/ml solution, IP administration of 20 mg per 100 g weight). After euthanasia, the rats were perfused transcardially with 0.9% saline followed by 4% paraformaldehyde. The brains were then removed and fixed in 10% buffered formalin for 48 h at room temperature. Following fixation, the samples were processed through graded ethanol, cleared with xylenol, and embedded in paraffin. Serial (5-µm-thick) coronal sections were obtained using a microtome and stained with hematoxylin and eosin (H&E) [20]. For this study, the parietal cortex and the hippocampal CA1 region were the primary areas of interest. The hippocampal sections were specifically taken at the following coordinates: −4.56 mm from Bregma, 3.5 mm depth, and 3.5 mm laterality, targeting the dorsal hippocampus.

### TTC staining

To assess the volume of brain lesion, TTC (Sigma-Aldrich, USA) staining was performed. Using a brain matrix, the brains were sliced into 2 mm-thick coronal sections. The slices were incubated in a 2% TTC solution prepared in distilled water at 37 °C for 20 min. After staining, the slices were fixed in 10% buffered formalin for 24 h to preserve the tissue structure. Following fixation, digital images of each slice were captured using a high-resolution camera (Cannon, Japan). Then, the lesion volume (mm^3^) was quantified using NIH Image Analyzer software.

### Brain water content

The following formula was used to measure brain edema based on previous studies [21].

 $$\text{Brain edema percentage (brain water content)}=\frac{\textit{wet}\mathit\;\textit{brain}\mathit\;\textit{weight-dry}\mathit\;\textit{brain}\mathit\;\textit{weight}}{\textit{wet}\mathit\;\textit{brain}\mathit\;\textit{weight}}\times\;100$$

### Statistical analysis

Regarding the statistical test, if the distribution of the data was normal, to compare the quantitative variables between the tested groups, if the assumptions were met, first, to check the difference between the groups, repeated-measures ANOVA was used; then, one-way ANOVA was used. If necessary, the Newman Coles post hoc test was used. For two-sided tests, Tukey’s post hoc test was used if necessary. A significance level of 0.05 was used, and statistical analyses were performed via GraphPad Prism 8 software. All the data are presented as the means ± SEMs. In all cases, a significance level of 0.05 (*P* ≤ 0.05) was considered.

## Results

### QC improved neurological function in MCAO rats

**Fig. 1:**
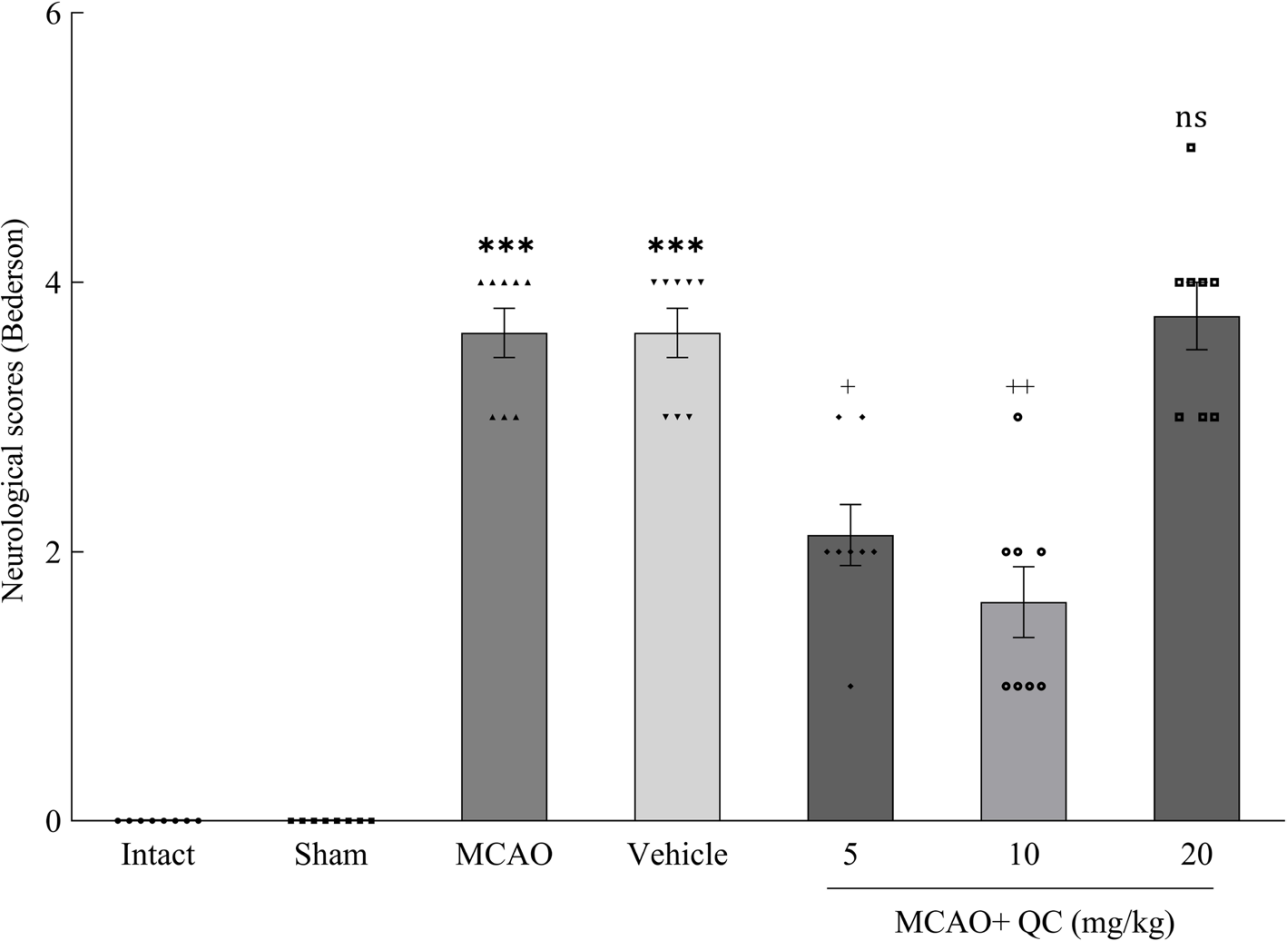
Bederson neurologic scores in different study groups (*** *P* < 0.001, + *P* < 0.05, ++ *P* < 0.01, ns: not significant)

Compared with the Intact and Sham groups, the MCAO and Vehicle groups had significantly higher Bederson scores (*P* < 0.001). Compared with those of the MCAO and vehicle groups, the Bederson scores of the groups receiving low and medium doses of QC were significantly lower (*P* < 0.05 and *P* < 0.01, respectively). However, the high-dose group showed no significant difference (Fig. 1).

### QC improved motor function in MCAO rats

**Fig. 2:**
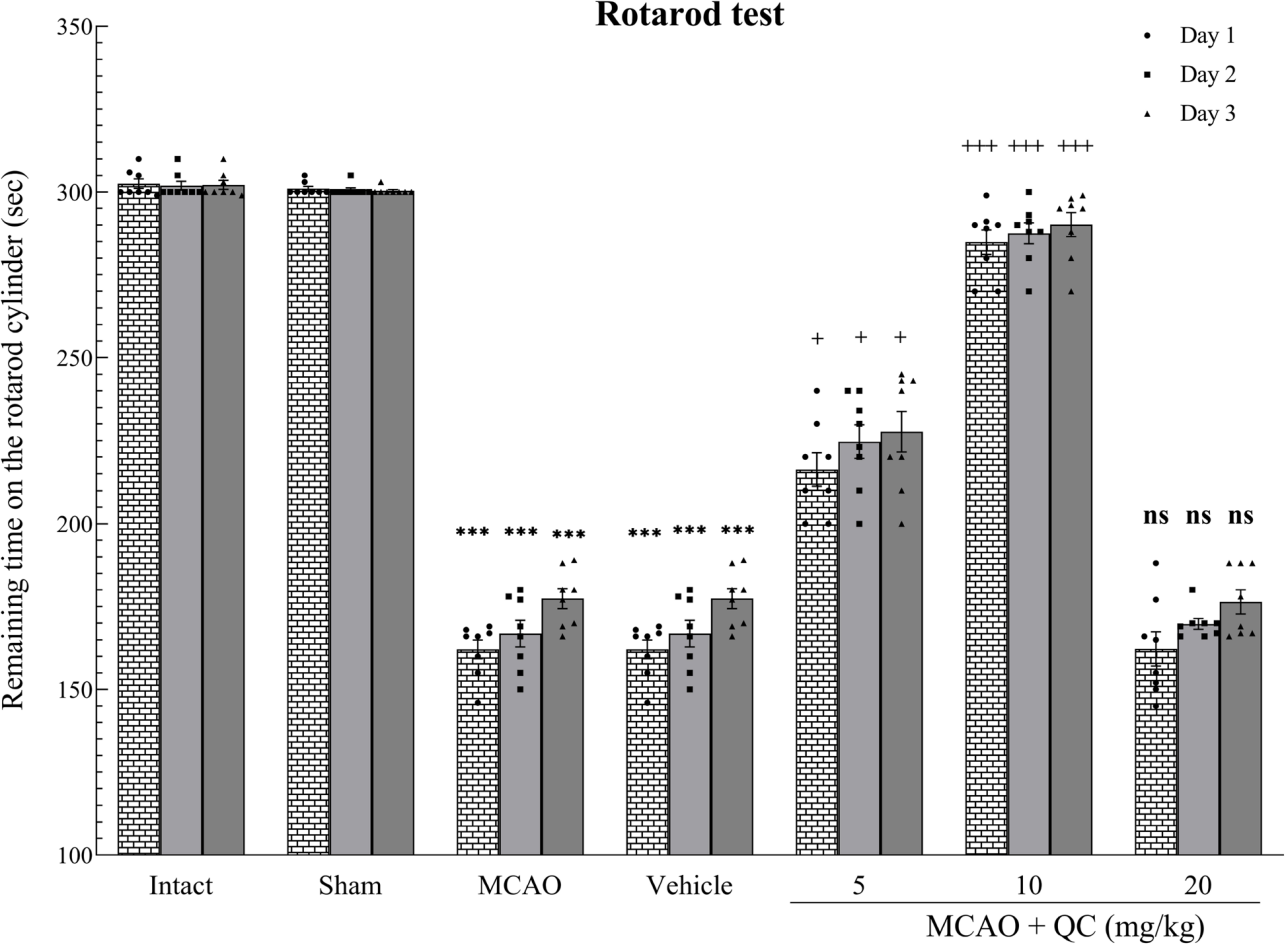
The time rats remained on the Rota-Rod device in different study groups (*** *P* < 0.001, + *P* < 0.05, +++ *P* < 0.001, ns: not significant)

Compared with those in the MCAO and vehicle groups, the number of rats that remained on the rotarod device was significantly greater in the groups that received low and medium doses of QC (*P* < 0.05 and *P* < 0.001, respectively). However, the high-dose group showed no significant difference (Fig. 2).

### QC improved spatial memory in MCAO rats

**Fig. 3:**
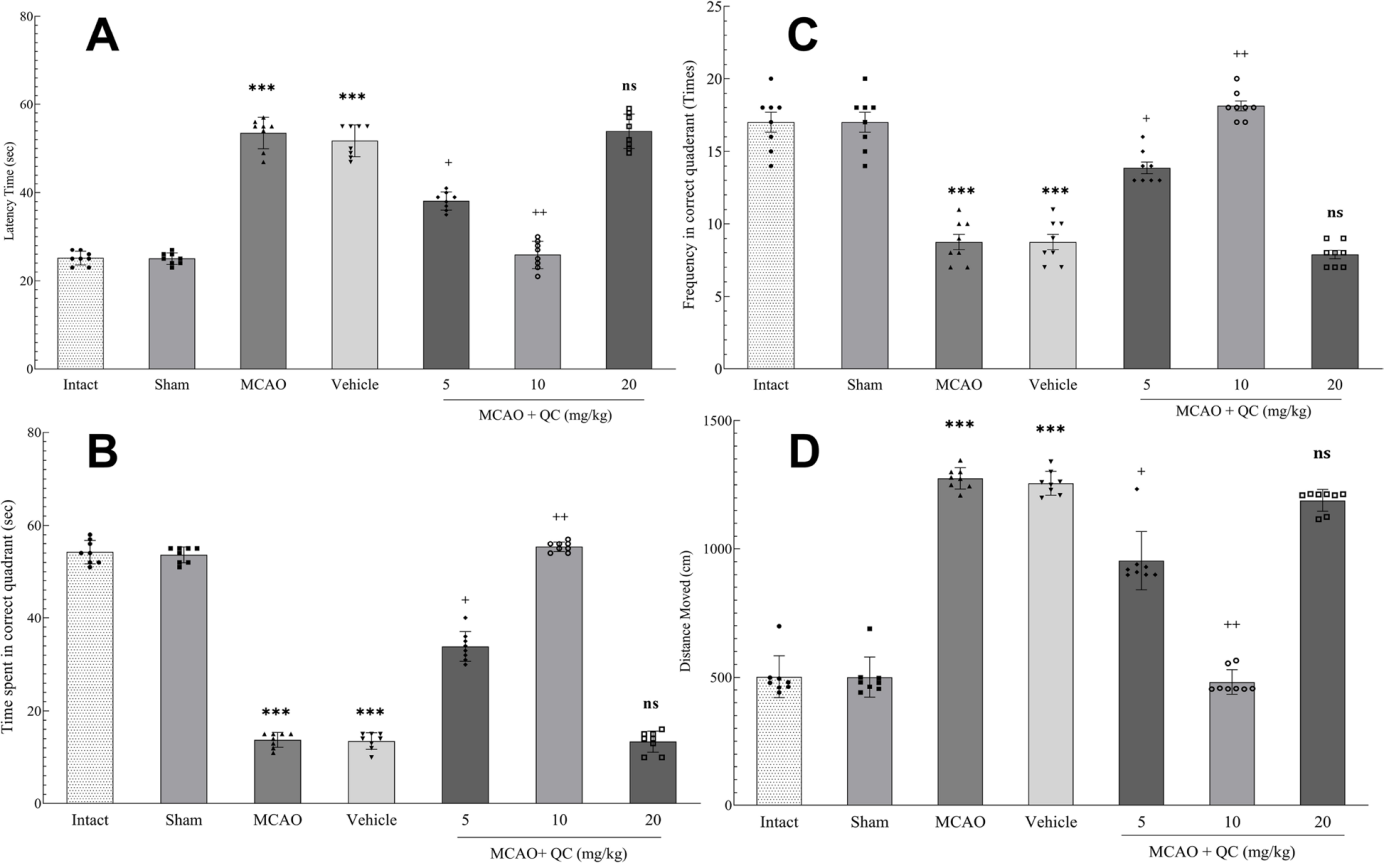
MWM test results. **A** The latency time for rats to reach the platform; **B** The time duration spent in the correct quadrant; **C** the frequency of times within the correct platform; and **D** the distance covered to reach the platform (*** *P* < 0.001, + *P* < 0.05, ++ *P* < 0.01, ns: not significant)

The MWM test results revealed that, compared with those in the MCAO and vehicle groups, the latency time for the rats to reach the platform, the time spent in the correct quadrant, the frequency of times spent within the correct quadrant, and the distance traveled to reach the platform were significantly greater in the low- and medium-concentration groups (Fig. 3). However, the high-dose group showed no significant difference.

### QC improved PAL in the shuttle box device

**Fig. 4:**
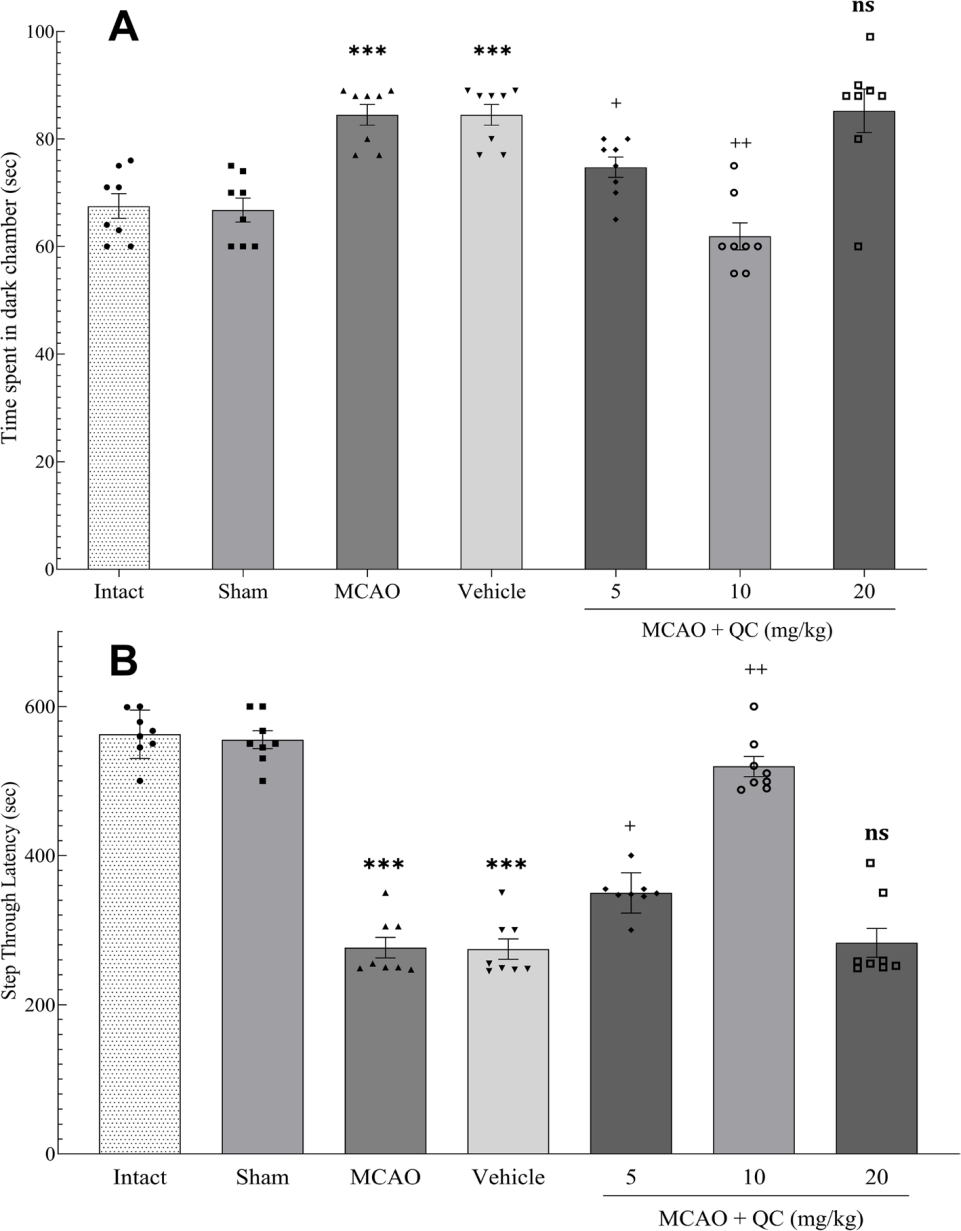
Shuttle box test results. **A** The time spent in the dark chamber; **B** The time duration spent in the light chamber or STL (*** *P* < 0.001, + *P* < 0.05, ++ *P* < 0.01, ns: not significant)

The number of acquisition trials did not differ between the study groups. Our results revealed that the low- and medium-dose groups presented significant improvements in both the STL and the time spent in the dark chamber. However, the high-dose group showed no significant improvements (Fig. 4).

### QC increased IL-10 and decreased IL-1β in the CSF

**Fig. 5:**
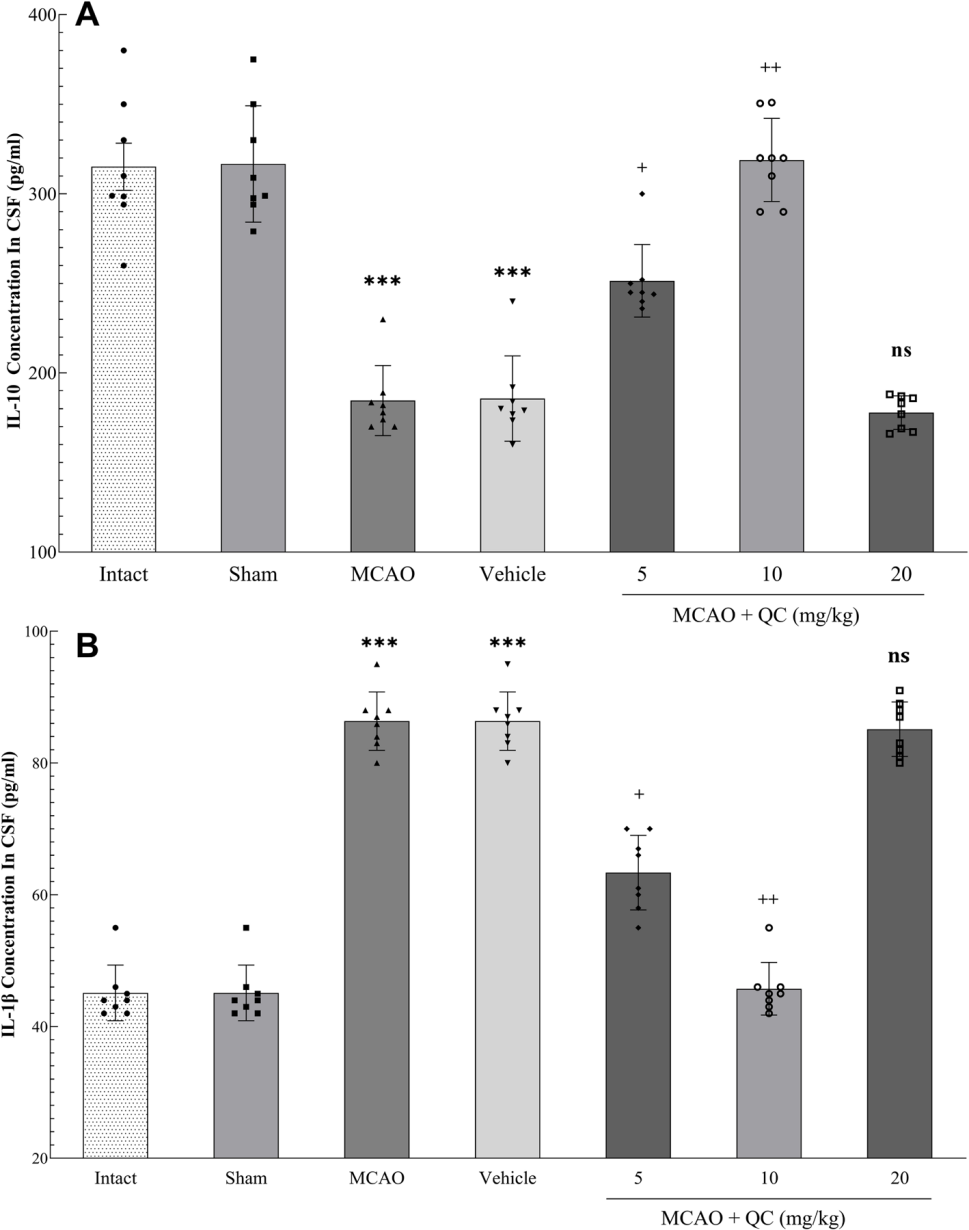
Concentration of interleukins in the CSF. **A** Concentration of IL-10 in the CSF; **B** Concentration of IL-1β in the CSF (*** *P* < 0.001, + *P* < 0.05, ++ *P* < 0.01, ns: not significant)

Our results revealed that the concentration of IL-10 was significantly greater in the low-dose and medium-dose groups than in the MCAO and vehicle groups, whereas the concentration of IL-1β was significantly lower; however, no increase in both IL-10 and IL-1β was detected in the high-dose group (Fig. 5).

### QC improved brain edema and brain lesion volume in MCAO rats

**Fig. 6:**
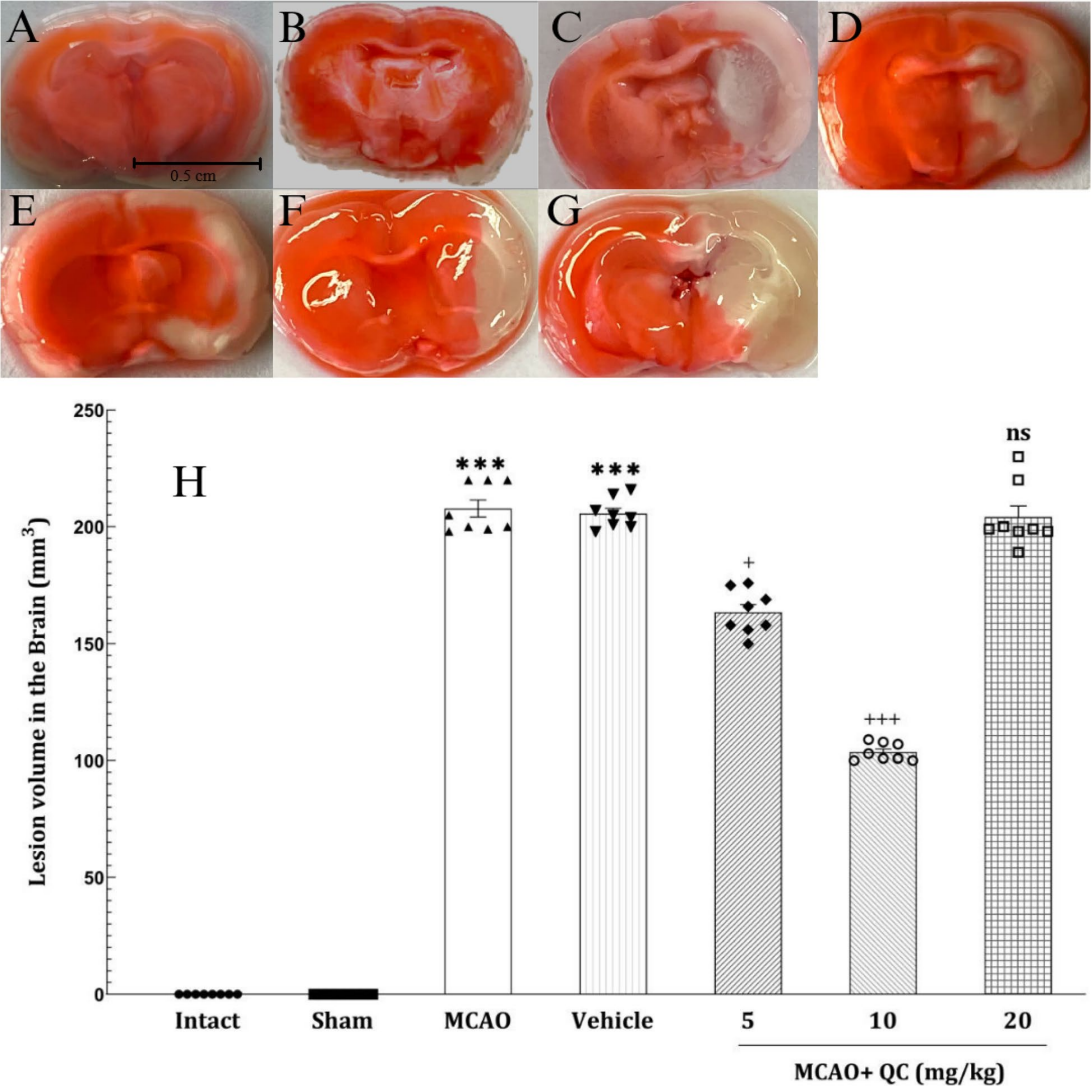
TTC stained brain coronal slides in **A** (intact group), **B** (sham group), **C** (Solvent group), **D** (Stroke group), **E** (QC 5 mg/kg), **F** (QC 10 mg/kg), and **G** (QC 20 mg/kg). Ischemic regions appear white and non-ischemic regions are stained red. **H** shows the significant reduction in brain lesion volume in QC 5 and 10 mg/kg treated groups. (*** *P* < 0.001, + *P* < 0.05, +++ *P* < 0.001, ns: not significant)

**Fig. 7:**
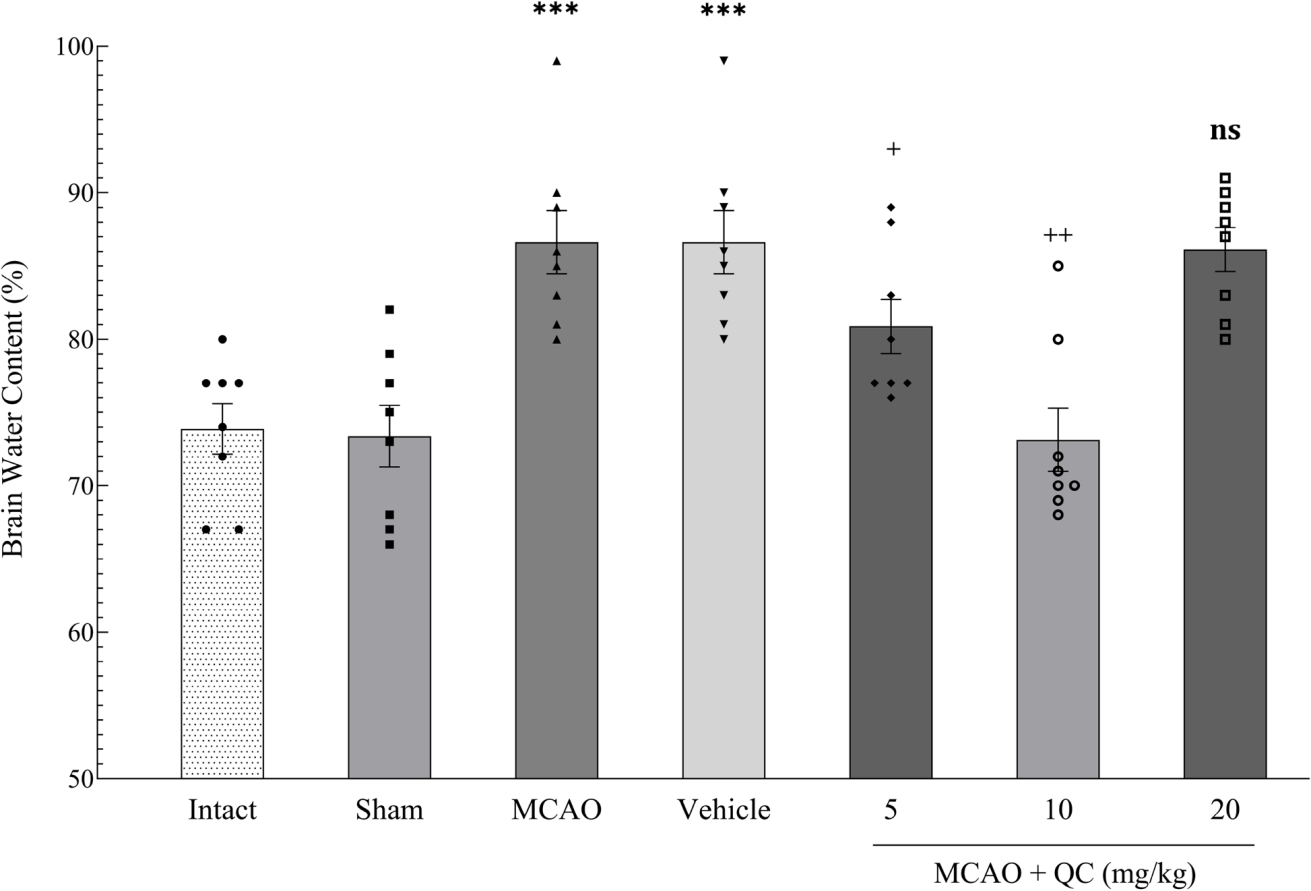
Brain water content in different study groups (*** *P* < 0.001, + *P* < 0.05, ++ *P* < 0.01, ns: not significant)

The brain lesion volume (measured by TTC staining) was significantly greater in the MCAO and vehicle groups than in the Intact and Sham groups (*P* < 0.001), indicating the successful MCAO induction (Fig. 6). Also, the brain water content was significantly greater in the MCAO and vehicle groups than in the Intact and Sham groups (*P* < 0.001), indicating that the brain water content increased after stroke induction. In the groups receiving low and medium doses of QC, the brain water content was significantly lower (*P* < 0.05 and *P* < 0.01, respectively), with the medium dose having the greatest protective effect on this matter. However, the group receiving high-dose QC was not significantly different from the vehicle group (Fig. 7).

### QC tends to decrease neural apoptosis

**Fig. 8:**
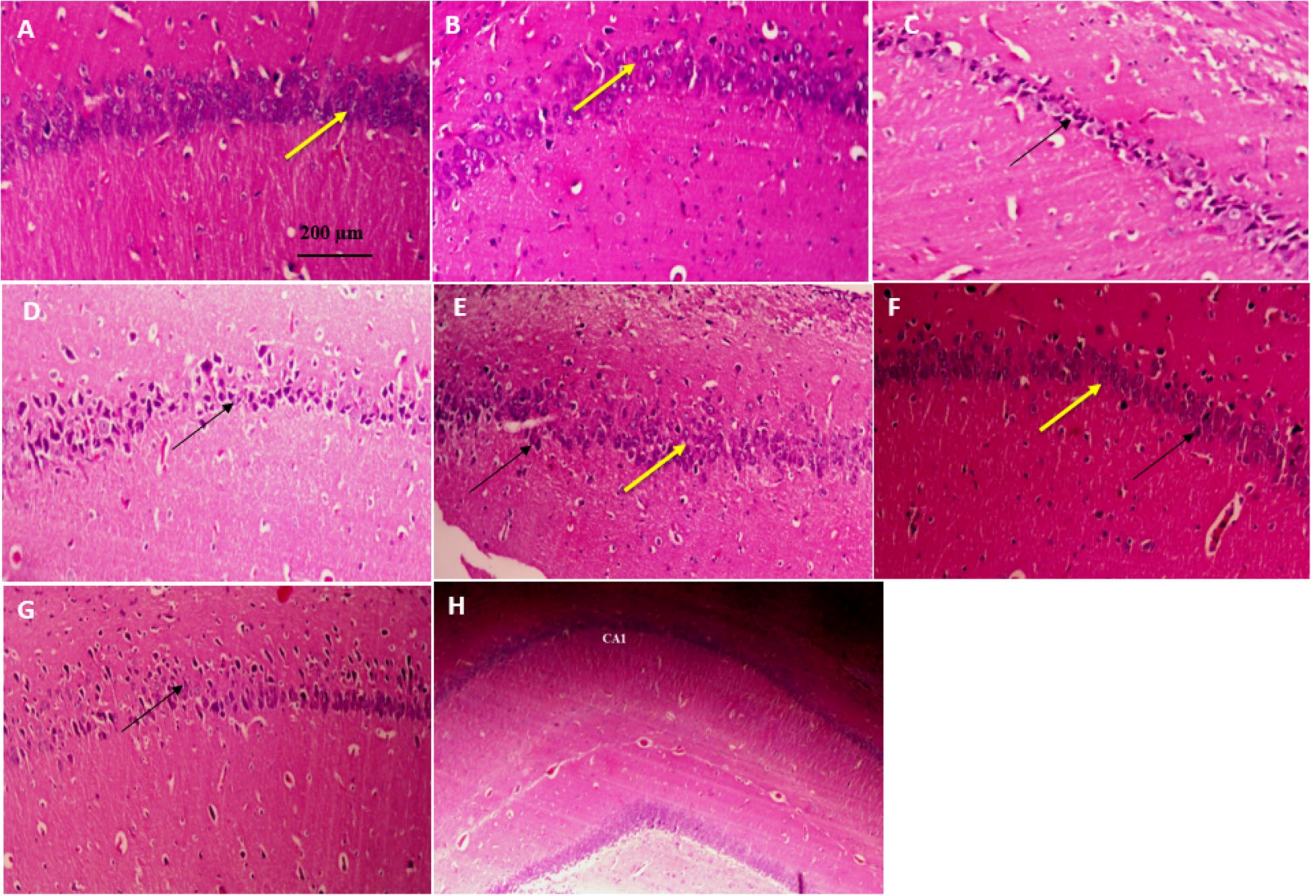
This is the representative photomicrograph of the hippocampus which shows the effect of QC treatment on CA1 region (H&E staining, magnification 400x). **A** Intact; **B** Sham; **C** MCAO; **D** Vehicle; **E** QC 5 mg/kg; **F** QC 10 mg/kg; **G** QC 20mg/kg ; **H** CA1 region (magnification 40x). Yellow arrow indicates normal neurons; Black arrow indicates apoptotic neurons. In the control groups [Intact (**A**), sham (**B**)]: pyramidal cells with round nuclei, prominent nucleoli and clear cytoplasm are visible. In MCAO and Vehicle groups [(**C**), (**D**)]: apoptotic cells with highly dense, granular and wrinkled nucleus, indistinct nuclei and cytoplasm are observed with increased staining. In treatment groups: **E **In 5 mg/kg dose, normal cells with clear nuclei are observed along with damaged cells with granulated and pigmented nuclei. **F** In 10 mg/kg dose, normal cells with clear nuclei are observed along with a few damaged cells with granulated and pigmented nuclei. **G** In 10 mg/kg dose, apoptotic cells with highly dense, granular and wrinkled nucleus, indistinct nuclei and cytoplasm are observed with increased staining. **H** Overview of the hippocampus and the CA1 area

As illustrated in Fig. 8, the groups without stroke (A, B) exhibit normal cells characterized by clear nuclei and an absence of apoptotic features. In contrast, following the stroke, groups C and D display cells with prominent apoptotic characteristics and hyperpigmentation. Upon administration of QC treatment, neuronal recovery is observed, with groups receiving 5 mg/kg and 10 mg/kg showing cells that tend to have a normal morphology and clear nuclei, although a small number of apoptotic cells are still present. However, at the higher dose of 20 mg/kg, no significant changes are noted, suggesting that QC treatment at this dose does not exert a noticeable effect.

## Discussion

The present study had aimed to investigate the neuroprotective effects of QC on hippocampal CA1 neurons in a rat model of MCAO. Our findings showed that QC (at 5 and 10 mg/kg dosages) can result in better neurological function, less infarction volume, less brain edema, and better histological outcomes while at higher doses (20 mg/kg), these neuroprotective effects may be reversed.

The neuroprotective effects of QC may involve the modulation of several key molecular pathways. For example, it is known to inhibit the NF-κB pathway, a critical regulator of inflammation and cell survival [22]. By suppressing NF-κB activation, QC can reduce the production of proinflammatory cytokines such as IL-1β, thereby protecting neuronal cells from inflammation-induced damage [23]. Additionally, the PI3K/Akt pathway, which promotes cell survival and prevents apoptosis, may be upregulated by QC, offering further protection against ischemic damage [24, 25]. Additionally, the lack of effect at higher doses of QC might be due to a saturation of its bioavailability or potential adverse effects at higher concentrations [26, 27]. It is possible that higher doses could lead to toxicity or downregulation of protective pathways, negating the benefits observed at moderate doses [26, 27]. While QC shows neuroprotective properties at moderate doses, high doses may exceed its optimal therapeutic window, leading to potential neurotoxicity. Excessive doses of antioxidants like QC can paradoxically cause pro-oxidant effects, resulting in the generation of reactive oxygen species (ROS) and subsequent oxidative stress. This phenomenon, known as the antioxidant paradox, could explain the reversal of neuroprotective effects at higher doses [28]. Also, QC may interact with other bioactive compounds or endogenous molecules in the brain. At higher concentrations, QC could bind to multiple protein targets non-specifically, altering signaling pathways and reducing its therapeutic specificity. Specifically, interactions with endogenous antioxidants like glutathione may lead to depletion of these critical molecules, diminishing the overall antioxidant defense of the brain [29]. The observed effects of QC may follow a hormetic dose-response curve, where low to moderate doses elicit beneficial effects, but higher doses lead to adverse outcomes [30]. Hormesis is a common phenomenon in pharmacology and toxicology, particularly with natural compounds, where mild stress induced by a moderate dose promotes adaptive responses, but excessive stress from higher doses leads to cellular dysfunction [31]. Further pharmacokinetic studies are necessary to understand the optimal dosing range for neuroprotection.

In this study, the brain water content significantly increased after the induction of stroke. In the mice that received the QC, the brain water content decreased significantly. QC at moderate doses had the greatest neuroprotective effect, reducing the brain water content, whereas high doses did not have a significant effect in this regard. Additionally, in the evaluation of neurological disorders via the Bederson rating system, the QC showed a significant improvement in neurological function. Moreover, a moderate dose of QC had the best neuroprotective effect, whereas high doses did not have a notable effect on reducing the Bederson neurological score. Consistent with our study, Park et al. [32] reported that QC significantly reduced brain water content and prevented neurological dysfunction. Dong et al. [33] studied the effects of QC on neural damage in mice with subarachnoid hemorrhage (SAH). They reported a significant reduction in brain edema in SAH mice receiving QC, which aligns with our study’s results. Our findings are consistent with those of Park et al. [32] and Dong et al. [33], who reported a significant reduction in brain water content and neurological deficits with QC treatment. However, unlike these studies, our high-dose QC group did not show significant improvements, suggesting a possible saturation point or dose-related toxicity that requires further investigation.

In the evaluation of spatial learning and memory via the MWM test, we found that mice receiving low and moderate doses of QC reached the hidden platform in a significantly shorter time and distance. QC also increased the time and frequency of the rat’s presence in the correct quadrant, which was statistically significant, with the moderate dose being the most effective. High doses of QC did not cause significant changes in any of the mentioned parameters. In a study by Lei et al. [34], mice subjected to brain ischemia/reperfusion injury presented learning and spatial memory issues compared with those in the sham group, with the former reaching the target quadrant later and spending less time there. However, in the QC group, the mice reached the target quadrant significantly sooner and spent more time there. Lei et al. [34] concluded that the inhibition of brain damage due to ischemia/reperfusion by QC probably involves a transcriptional mechanism to enhance antiapoptotic signaling. Similarly, Le et al. [35] reported that QC improved cognitive functions in mice with ischemic brain injury. They demonstrated through the MWM test that QC treatment led to improvements in both the duration and frequency of access to the target area. Since hippocampus-dependent cognitive functions are evaluated via the MWM test, as shown in this and other studies, it can be assumed that QC may improve the performance of this brain region.

To assess PAL and memory, a shuttle box device was used. Initially, the number of learning sessions required for the mice was recorded, and comparisons revealed that the QC did not affect the number of sessions needed for learning in any of the groups. However, in the evaluation of PAL, QC reduced the time spent in the dark chamber. As before, low and moderate doses significantly reduced this time, with the moderate dose being more effective than the low dose in terms of hippocampal function. No studies have examined passive avoidance memory after QC treatment in mice with induced ischemia/reperfusion, but in a study by Kaedi et al. [36], which examined the effect of QC on mice with learning and memory deficits induced by lipopolysaccharide injection, QC was found to significantly increase the STL and improve passive avoidance memory. Singh et al. [37] studied zebrafish with neuroinflammation and oxidative stress induced by lipopolysaccharide and reported that QC injection caused the fish to spend less time in the dark compartment, significantly improving passive avoidance memory.

The assessment of balance and motor function in the rats was performed via a rotarod device. Compared with those in the sham group, the rats that did not receive QC after MCAO experienced balance and motor impairment. Low and moderate doses of QC in rats with induced cerebral ischemia improved their balance and motor function. As with the previous parameters, high doses of QC did not improve ischemic brain injury due to MCAO. Similarly, Lee et al. [38] reported that QC improved balance and motor function in mice with cerebral ischemia, and Lv et al. [39] reported that QC improved balance and motor function in Parkinsonian mice.

After ischemic injury, many cytokines are upregulated in immune cells, as well as in resident brain cells such as neurons and glia [40]. While some cytokines, such as IL-1, seem to exacerbate brain injury, others (such as IL-6, IL-10, and TGF-β) appear to provide neuroprotection [41–43]. The proinflammatory cytokine IL-1β is associated with neurodegenerative diseases caused by excitotoxic or traumatic injury, most notably in experimental cerebral ischemia in rodents [44, 45]. In the present study, IL-1β levels in the CSF of mice were measured and compared. Compared with the intact and sham groups, the MCAO and vehicle groups presented significantly higher IL-1β levels. Conversely, compared with those in the MCAO and vehicle groups, the IL-1β levels in the groups receiving low and moderate doses of QC were significantly lower. Additionally, comparisons revealed that IL-10 levels increased with low and moderate doses of QC, whereas high doses were ineffective. Similarly, Zhang et al. [46] reported that after MCAO, combination treatment with human umbilical cord mesenchymal stem cells (HUMSCs) and QC could reduce IL-1β and IL-6 levels and increase the levels of anti-inflammatory cytokines such as IL-10, IL-4, and TGF-β.

Activated microglia and astrocytes are responsible for releasing inflammatory factors such as IL-1β, causing neuroinflammation [47–49]. Several studies have reported increased IL-1β expression under pathogenic conditions, brain injury, and degeneration [50, 51]. Since QC significantly reduces IL-1β in the brain, it can decrease brain injuries caused by proinflammatory cytokines. QC, a natural flavonoid, can reduce brain damage caused by stroke, especially in the CA1 region of the hippocampus. This protective effect of QC can be attributed to various mechanisms. In a study by Chen et al. [4], the protective effect of QC on CA1 pyramidal neurons of the hippocampus was observed and confirmed by immunohistochemistry of neuronal nuclear antigen and Fluoro-Jade B histofluorescence staining. Pei et al. [52] demonstrated that QC significantly reduced cognitive impairments in the MWM test, reducing apoptosis and neuronal death by inhibiting the ASK1/JNK3/caspase-3 pathway and inducing the Akt signaling pathway. Ming-Cheng Lin et al. [53] reported that QC could reduce oxidant factors and increase the levels of antioxidants such as catalase and superoxide dismutase (SOD). Therefore, it is evident that QC can exert its protective effect on neuronal cells through various mechanisms.

Brain injury induces the production of reactive oxygen species (ROS) and oxidative stress, which damage cellular components. As a potent antioxidant, QC can scavenge free radicals and diminish oxidative stress. Furthermore, QC enhances the activity of endogenous antioxidant enzymes such as SOD, catalase, and glutathione peroxidase, providing additional protection to brain cells from oxidative damage. Brain injury often leads to disruption of the blood‒brain barrier (BBB), resulting in edema and further injury. QC has been demonstrated to protect and preserve BBB integrity, thereby reducing edema and secondary injury. Moreover, QC can inhibit pathways that lead to neuronal apoptosis. It modulates critical signaling pathways, such as the PI3K/Akt and MAPK pathways, which are vital for preventing cell survival and apoptosis. By maintaining mitochondrial function, which is crucial for energy production and cell survival, QC supports neuronal health and function. QC also inhibits the JAK-STAT signaling pathway, which is implicated in inflammation and cell death following brain injury. This inhibition reduces inflammation and promotes cell survival. Additionally, QC suppresses the nuclear factor kappa B (NF-κB) pathway, which is activated in response to trauma and induces the expression of proinflammatory genes. By inhibiting NF-κB activation, QC further reduces inflammation and tissue damage. The effectiveness of QC in the treatment of brain injury is attributed to its multifaceted actions, including anti-inflammatory, antioxidant, and neuroprotective effects. By modulating key signaling pathways and reducing inflammation and oxidative stress, QC protects brain cells, preserves BBB integrity, and enhances functional recovery after brain injury. While preclinical studies are promising, further research, including clinical trials, is necessary to fully establish the therapeutic potential of QC in human brain injury.

### Strengths and limitations

This study’s combination of behavioral, biochemical, and histological assessments provides a holistic understanding of the effects of QC. It stands out for its ability to connect improvements in neurological function with changes at the cellular and molecular levels (e.g., IL-1β and IL-10 modulation). This integrated approach enhances the robustness of the findings, offering both mechanistic insights and practical implications. Unlike previous studies that investigated the effects of QC in global cerebral ischemia or neurodegeneration models, this study focused on the hippocampal CA1 region following focal ischemia‒reperfusion injury induced by MCAO​. This specificity in targeting regional ischemia in the brain provides novel insight into how QC protects one of the most vulnerable areas affected by ischemic stroke. Although this study effectively demonstrated the benefits of QC in a rat model, the direct translation of these findings to humans remains uncertain. Animal models often exhibit different pharmacodynamic responses, and further research, including clinical trials, is needed to evaluate the efficacy of QC in human ischemic stroke patients.

Also, while our findings indicate an association between quercetin treatment and altered expression levels of IL-10 and IL-1β, these results do not confirm a causal role of these cytokines in mediating neuroprotection. Future studies employing targeted approaches, such as IL knockdown or overexpression models, are required to elucidate the direct involvement of these factors in quercetin’s neuroprotective mechanism.

## Conclusion

QC can prevent various brain injuries caused by stroke and even improve them in some cases, thus exerting its neuroprotective effect. Also, in the present study, we showed that the reduction in inflammatory factors such as IL-1β and, in contrast, the increase in anti-inflammatory cytokines such as IL-10 could be vital factors in the neuroprotective role of QC.

## Data Availability

The data are available upon reasonable request from the corresponding author.
